# Comparison of Different Thermo-Chemical Treatments Methods of Ti-6Al-4V Alloy in Terms of Tribological and Corrosion Properties

**DOI:** 10.3390/ma13225192

**Published:** 2020-11-17

**Authors:** Jacek Grabarczyk, Damian Batory, Witold Kaczorowski, Bartosz Pązik, Bartłomiej Januszewicz, Barbara Burnat, Małgorzata Czerniak-Reczulska, Marcin Makówka, Piotr Niedzielski

**Affiliations:** 1 Institute of Materials Science and Engineering, Lodz University of Technology, 1/15 Stefanowskiego St., 90-924 Lodz, Poland; witold.kaczorowski@p.lodz.pl (W.K.); bartoszpazik@gmail.com (B.P.); bartlomiej.januszewicz@p.lodz.pl (B.J.); malgorzata.czerniak-reczulska@p.lodz.pl (M.C.-R.); marcin.makowka@p.lodz.pl (M.M.); piotr.niedzielski@p.lodz.pl (P.N.); 2 Department of Vehicles and Fundamentals of Machine Design, Lodz University of Technology, 1/15 Stefanowskiego St., 90-924 Lodz, Poland; damian.batory@p.lodz.pl; 3 Department of Inorganic and Analytical Chemistry, University of Lodz, 12 Tamka St., 91-403 Lodz, Poland; barbara.burnat@chemia.uni.lodz.pl

**Keywords:** titanium alloys, thermo-chemical treatments, carburizing, nitriding, oxidation, tribological properties, corrosion properties

## Abstract

Titanium and its alloys are characterized by high mechanical strength, good corrosion resistance, high biocompatibility and relatively low Young’s modulus. For many years, one of the most commonly used and described titanium alloys has been Ti-6Al-4V. The great interest in this two-phase titanium alloy is due to the broad possibilities of shaping its mechanical and physico-chemical properties using modern surface engineering techniques. The high coefficient of friction and tendency to galling are the most important drawbacks limiting the application of this material in many areas. In this regard, such methods as carburizing, nitriding, oxidation, and the synthesis of thin films using physical vapor deposition (PVD) and chemical vapor deposition (CVD) methods may significantly improve the tribological properties of titanium alloys. The influence of thermo-chemical treatment (oxidation, carburizing and nitriding) on tribological properties and corrosion resistance of Ti-6Al-4V alloy is presented in this paper. The results include metallographic studies, analysis of tribological and mechanical properties and corrosion resistance as well. They indicate significant improvements in mechanical properties manifested by a twofold increase in hardness and improved corrosion resistance for the oxidation process. The carburizing was most important for reducing the coefficient of friction and wear rate. The nitriding process had the least effect on the properties of Ti-6Al-4V alloy.

## 1. Introduction

Titanium and its alloys are very attractive materials for many engineering applications. They are used, among others, in the aerospace, automotive, energy, shipbuilding, chemical and food industries, as well as in medicine [1,2,3,4]. Ti-6Al-4V alloy is the most commonly used and widely described in the literature. This two-phase material is characterized by low specific weight, high mechanical strength and a Young’s modulus of less than half that of steel. Ti-6Al-4V is also highly biocompatible and corrosion-resistant [5,6]. On the other hand, this material has poor tribological properties, as demonstrated by the high coefficient of friction in combination with most of the materials, tendency for scuffing and adhesive wear as well [6,7]. Literature reports confirm that the heat treatment and alloying additives do not contribute to the improvement of these unfavorable properties [6,8]. Researchers in many centers in the world have confirmed the beneficial effects of thermo-chemical methods (nitriding, carburizing, oxidation) and the synthesis of thin films using physical vapor deposition (PVD) and chemical vapor deposition (CVD) methods on tribological properties of titanium alloys. In this field, the significant effect of the nitriding process on the increase in hardness of the surface of the Ti-6Al-4V alloy was reported [9,10,11]. The authors showed that the process parameters had a significant impact on the obtained results. The increase in temperature and time of the process ensured a thicker nitrogen-saturated layer, resulting in greater hardness and abrasion resistance. Often, apart from conventional gas nitriding, this process may be assisted by glow discharge, which allows to one obtain good tribological properties at lower modification temperatures [9]. As shown by Batory et al. [12], radio frequency plasma nitriding at 580 °C makes it possible to obtain a very high resistance of Ti-6Al-4V alloy against liquid impingement erosion just after 4 h of the process. Nowadays, there are many works presenting the oxidation process as a hardening treatment, of which the main task is to improve the mechanical and tribological properties of the substrate. Another favorable result of the oxidation process is the increase in corrosion resistance due to the increased thickness and tightness of the oxide layer on the surface of the modified titanium and its alloys [13,14,15]. The most common methods for the oxidation of titanium alloys are modifications in a fluidized bed by gas and plasma techniques. The modification in a fluidized bed gives relatively low thickness of the oxygen-saturated layer of approximately several micrometers [14,15]. In the case of the gas oxidation assisted by plasma discharge, the obtained depth of diffusion layer may reach from 85 to 105 µm [16,17]. The carburization of titanium alloys is much less frequently described in the literature; however, some papers supported its beneficial effect on the tribological properties. The authors of ref. [18] achieved a 128% increase in hardness on the surface of Ti-6Al-4V alloy after the carburization process and higher fatigue strength. In addition, in works [19,20], the authors showed a significant increase in hardness and wear resistance. This, in turn, makes the carburizing of titanium alloys a very good alternative to the widely discussed nitriding and oxidation processes in the direction of improving their poor tribological properties.

It seems that in the case of medical titanium alloys, the thermo-chemical treatment is not enough to ensure favorable tribological properties [8]. The surface prepared in this way can, however, be an excellent basis for the synthesis of low-friction coatings, e.g., diamond-like carbon (DLC) [21,22]. These coatings require a pre-hardened substrate to ensure durability in highly loaded friction nodes. In the case of titanium alloys, the elastic/plastic deformation of the susceptible, unmodified substrate is most often responsible for the accelerated wear of hard and abrasion-resistant coatings [23,24]. This wear occurs as a result of deformation of the substrate below the layer, as a consequence of which the coating itself loses its stable support and cracks. One of the objectives of the research presented in this paper was to determine whether the tested thermo-chemical treatment processes (oxidation, carburization, gas nitriding) are sufficient to ensure good mechanical and tribological properties of titanium alloys while maintaining their appropriate corrosive properties. An additional goal was to check which of the investigated treatments enables strengthening of the titanium alloy to be a sufficient basis for the synthesis of low-friction protective coatings.

## 2. Materials and Methods

### 2.1. Material

The tested material was two-phase Ti-6Al-4V titanium alloy (Medgal Orthopaedic Implants and Instruments, Białystok, Poland) with chemical composition in agreement with the ISO 5832-2 standard. Initially, the material was annealed at 740 °C for a period of 120 min and cooled with the furnace.

Flat discs 14 and 25.4 mm in diameter and 6 mm high were used as samples for corrosion tests and tribological and mechanical characterization, respectively. The samples were ground and mirror polished using colloidal silica suspension. Before each thermo-chemical treatment, the substrates were ultrasonically cleaned in an acetone bath for 10 min and dried using a compressed air.

### 2.2. Thermochemical Treatment

The prepared samples were subjected to three independent thermo-chemical treatment processes. The oxidation of titanium alloy was carried out by means of glow discharge in Ar + O_2_ atmosphere in a vacuum furnace. The vacuum furnace was equipped with a quartz tube and specimens’ holder (tungsten electrode) mounted in the tube and connected to medium frequency power supply. The samples were mounted on the specimens’ holder and the quartz tube was pumped down to a pressure approx. 1 Pa. Pure Ar with flow of 30 sccm was introduced into the tube, the glow discharge power supply was turned on, and the furnace was heated. When the temperature in the quartz tube reached 900 °C, pure oxygen was introduced in a cycle mode (60 s of oxygen supply and 600 s of discharge without oxygen flow) and with flow equal to 1/6 of Ar flow. During the whole process, the power of discharge was kept at the same level and with voltage and current equal to 850–1000 V and 50–60 mA, respectively. The whole process consisted of 20 cycles of saturation of specimens’ surface with oxygen in a glow discharge with continuous Ar flow. After last cycle, furnace heating was turned off and the batch was slowly cooled down with the furnace. When the temperature inside the quartz tube was as low as room temperature, glow discharge power supply was turned off and specimens were unmounted from the tungsten electrode. The vacuum carburization processes were conducted in an atmosphere of acethylene, ethylene and hydrogen in a ratio of 1:1:2 at 950 °C for 0.5 to 2 h. The total flow of the carburizing mixture was 0.8 L/min. Carburizing processes were divided into two or more stages. In 1.5 h processes, it was 0.5 h of carbon surface saturation and then 1 h of annealing in vacuum (diffusion step). In the 2.5 h process, the first stage (saturation) lasted 0.5 h and the diffusion step lasted 2 h. After carburizing, the samples were automatically ejected into the cooling chamber. The cooling time to the temperature below 50 °C was about 240 s. The cooling process was conducted in nitrogen atmosphere.

The vacuum nitriding processes were carried out in high-purity ammonia gas (NH_3_) at 850 °C for 3 h. The pressure in the working chamber during the process was maintained at 30 mbar, with a working gas flow of 1 L/min. After the nitriding process, the samples were automatically ejected into the cooling chamber where they were cooled under elevated pressure of nitrogen. The cooling time to the temperature below 50 °C was about 240 s.

The parameters of all three thermo-chemical treatments presented above were selected based on the experience and preliminary authors’ own research.

### 2.3. Characterization Methods

Microstructural examination was carried out using the scanning electron microscope JEOL JSM-6610LV (JEOL Ltd.; 3-1-2 Musashino, Akishima, Tokyo 196-8558, Japan), equipped with the X-Max 80 X-ray microanalysis system. Structural analysis was performed for samples before and after the thermo-chemical treatment. Before the observation, the prepared cross sections were etched by Kroll’s reagent. Additionally, in the case of carburized samples, the Raman spectroscopy studies were performed using an inVia confocal micro-Raman spectrometer (Renishaw plc, Gloucestershire, UK), working with a 532 nm wavelength and a power of 2.5 mW.

The phase composition of the titanium alloy surface after low pressure nitriding was evaluated by the X-ray diffraction (XRD) method using Empyrean diffractometer (Panalytical Empyrean, Almeo, The Netherlands). The X-ray source was a Co tube worked at accelerating voltage of 40 kV and with a current value of 40 mA. A five axis X-Y-Z-Phi-Chi stage was used. The incident beam optics consisted of a parallel beam X-ray mirror for Co radiation with a 10 mm mask, an anti-scatter slit of 1.4 mm and Soller slits 0.04 rad. Scattered beam optics consisted of a parallel plate collimator of divergence 0.18 deg, Soller slits 0.04 rad and a proportional point Xe detector. The incident angle was 0.5 deg, with a scan range of 35 to 85 degrees 2Theta with 0.05 deg step size. The time per step was set to 5 s. Analysis of diffraction pattern was made with use of High Score Plus software (ver. 3.0 e, Panalytical Empyrean, Almeo, The Netherlands) and ICCD PDF 4+ database.

Mechanical properties were determined by the nanoindentation method with use of Nanoindenter G200 system (Agilent Technologies, Palo Alto, CA, USA). The nanoindentation tests were made using a diamond Berkovich tip (MicroStar Technologies, Huntsville, AL, USA). The tip shape was calibrated by a nanoindentation in a fused silica standard. Hardness distribution on the cross-sectional profile was obtained for a load of 10 mN. The tests were carried out at a strain rate of 0.05 s^−1^. The data were analyzed using the Oliver and Pharr approach [25].

Tribological parameters (coefficient of friction (CoF) and resistance against wear) were determined using ball on disc method. The investigations were performed using T-11M Tribometer (ITeE-PIB Radom, Radom, Poland) under load of 10 N with the sliding speed 0.1 m/s on a distance of 1000 m, as the counterbody ¼ inch in diameter ZrO_2_ (G5, Ra—0.02 µm) ball was used. The tests were performed at a temperature of 20 ± 1 °C and relative humidity of 50 ± 2%. After the tests, both the wear tracks on the samples and wear scars on the counterbodies were analyzed using the profilometer and scanning electron microscope to determine their specific wear rate. For each sample, four measurements were registered, and the results were averaged.

Corrosion tests were performed in phosphate buffered saline (PBS) solution composed of: 8 g/L NaCl, 0.2 g/L KCl, 0.2 g/L KH_2_PO_4_ and 2.9 g/L Na_2_HPO_4_·12H_2_O in distilled water (pH 7.4 was adjusted with HCl) [26]. The PBS solution was maintained at a temperature of 37 °C, and its degassing was achieved by argon bubbling throughout all corrosion tests. The electrochemical cell set up for the corrosion experiments was of the three-electrode type (400 mL Corrosion Cell, Methrom Autolab) with a platinum rod as a counter electrode, a saturated calomel electrode (SCE, Elmetron, Zabrze, Poland) as a reference electrode, and a tested specimen (with an exposed area of 0.785 cm^2^) as a working electrode An Autolab potentiostat PGSTAT 302N operated by NOVA 1.11 software (Metrohm Autolab B.V., Utrecht, The Netherlands)was used for the electrochemical measurements. Prior to the beginning of the polarization tests, the specimens were kept in the solution for 1800 s in order to establish the free corrosion potential (E_cor_). Two types of polarization test were performed in order to determine corrosion properties of investigated samples. Polarization in a narrow potential range (±20 mV vs. E_cor_) with scan rate of 0.3 mV/s allowed us to determine the values of polarization resistance R_p_ and corrosion current density i_cor_, according to Stern-Geary’s method, and thus the corrosion rate value could be calculated according to the equation:

$$\mathrm{CR}=K_{1}\frac{i_{\mathrm{cor}}}{\rho }\mathrm{EW}$$

where: CR—corrosion rate (mm/year), K_1_—coefficient (3.27·10^−3^ mm g/μA cm year), i_cor_—corrosion current density, determined from Stern-Geary’s characteristics (μA/cm^2^), ρ—density of Ti-6Al-4V alloy (4.43 g/cm^3^), EW—equivalent weight for Ti-6Al-4V alloy (11.88, calculated for elements above 1 mass% in the alloy).

In order to determine the resistance against pitting corrosion of the investigated samples, the polarization in wide anodic potential range (from 0.2 V below E_cor_ potential up to 4 V vs. SCE) was performed, and thus potentiodynamic curves were obtained.

Each experiment was repeated three times to ensure its reproducibility. The corrosion results are presented as mean values with standard deviations.

After corrosion tests, the sample surfaces were analyzed using scanning electron microscopy.

All tests were performed on three independent samples of each type and the results were averaged.

## 3. Results and Discussion

### 3.1. Scanning Electron Microscopy

**Figure 1:**
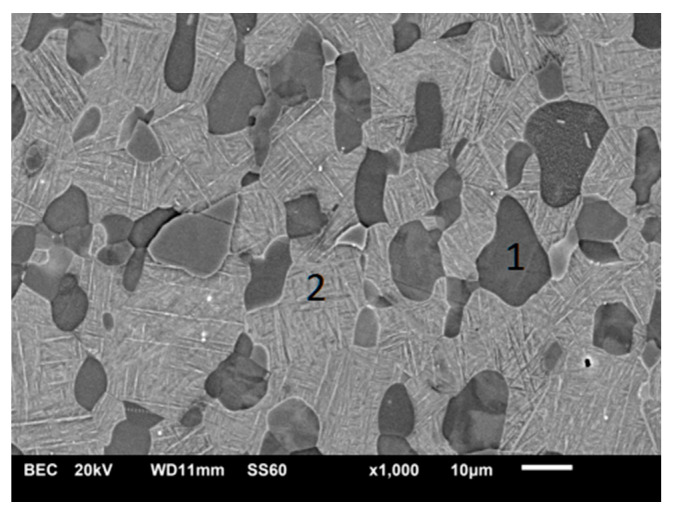
Bimodal microstructure of Ti-6Al-4V substrates. Phase marked 1—primary α phase, phase marked 2—transformed β phase.

The microstructure of unmodified Ti-6Al-4V alloy shows a bimodal character (Figure 1). Clear equiaxial α-Ti grains are visible against a mixture of β-Ti precipitations in α-Ti matrix. The microstructure of sample nitrided at 850 °C shows a very thin compound Ti-N-based layer on the top. This observation is in agreement with other literature reports, since during the vacuum nitriding processes, the formation of a Ti_2_N/TiN based-compound layer is not inhibited as compared to plasma nitriding [12,27].

**Figure 2:**
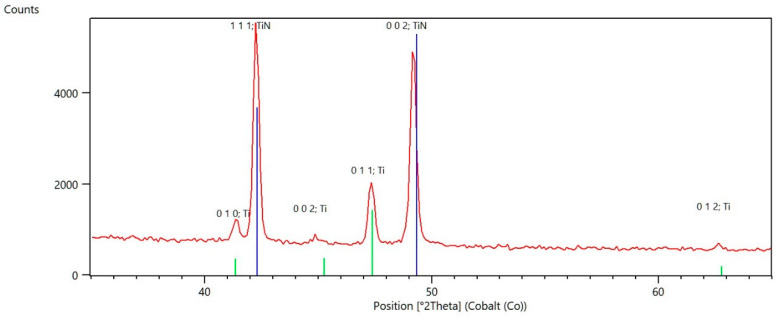
X-ray diffraction pattern for nitrided sample. Ti hexagonal alpha phase and surface layer of cubic TiN were detected.

**Figure 3:**
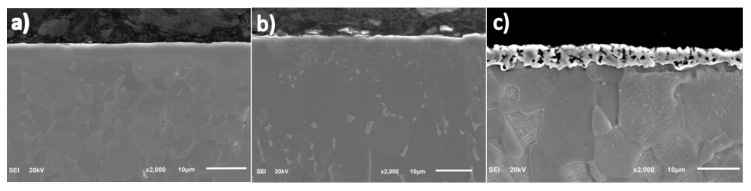
SEM images of the microstructure of the Ti6Al4V alloys after: (**a**) nitriding (**b**) oxidation (**c**) carburizing.

Moreover, during the XRD studies, a thin layer of TiN was registered on the surface (see Figure 2). Just beneath the surface, a thin continuous layer of nitrogen-stabilized α-Ti (α-case) is visible, which is followed by a diffusion zone (Figure 3a). These results are partially in agreement with those reported in ref. [28], where, besides the well pronounced Ti_2_N/TiN compound layer, the authors reported the same morphology of the nitrided layer. The difference may be caused by a slightly higher nitriding temperature (900 °C). For the sample oxidized at a temperature of 900 °C, a brighter area on the top may indicate the appearance of TiO_x_ compounds (Figure 3b). Next, a well-pronounced oxygen-stabilized α-Ti layer of considerable thickness and trace amounts of β-Ti are visible. Subsequently, the oxygen diffusion zone may be distinguished. The results are in agreement with work of Januszewicz et al. [19].

The process of carburizing resulted in the thickest compound layer on the top, mainly composed of TiC (Figure 3c). Here, the carbon stabilized α-Ti layer as well as the diffusion zone are barely noticeable, which may be caused by a rather low diffusion coefficient of carbon in titanium. Another possible reason for such a low thickness of the diffusion zone may be the formation of titanium carbide layer on the surface which, although very porous, effectively hinders the diffusion of carbon into deeper parts of the modified substrate. A distinct difference in grain size for the particular thermo-chemical treatments is worth mentioning.

### 3.2. Hardness

**Figure 4:**
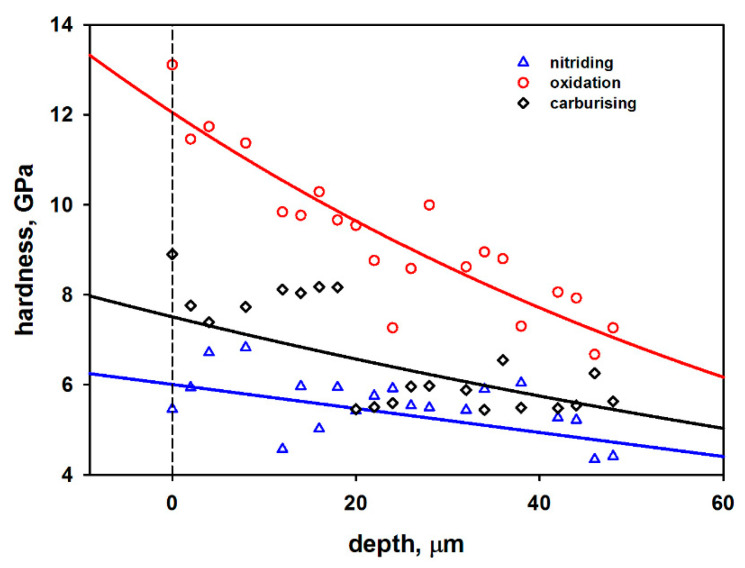
Hardness distribution on the cross-section of the thermochemically treated sample. The hardness is presented with respect to the distance from the surface of the sample.

The hardness distribution of the cross-section of the analyzed samples is presented in Figure 4.

A significant scatter of the results is probably caused by the geometry of the tip, which is small enough to also indent the grain boundaries [12,29]. Despite this fact, clear evidence of the positive influence of all three modification methods on internal strengthening of the modified samples is visible. The greatest range of diffusion and at the same time the highest strengthening of the top layer was obtained for the oxidation process. The registered hardness on the surface of the sample was ca. 12 GPa, whereas the thickness of the diffusion layer was more than 60 micrometers. This is also in good agreement with the results from SEM. The carburized layer reached a hardness of ca. 9 GPa. Here, the depth of the increased hardness region is relatively low and the trend of hardness distribution resembles a step-like shape. There is a lack of the characteristic hardness gradient and only the first 20 µm show the increased hardness. Moreover, for this first 20 µm, the value of hardness remains the same and amounts to ca. 8 GPa. Next, a sudden decrease in hardness is visible, wherein its value stays below 6 GPa and shows no noticeable variations. The lowest surface hardness and depth of the increased hardness region were registered for the nitrided samples. In this case, only for the few first indentations may slightly increased hardness be observed (hardness value slightly below 7 GPa), which confirms the results of the SEM investigation. Subsequently, the value of hardness drops below 6 GPa and similarly, as in case of the carburized sample, shows small variations. The surprisingly low and narrow hardness profile after nitriding is caused by the relatively low temperature and short time, as compared to ref. [28]. The electrical activation of the working atmosphere would increase the efficiency of the process, since the bombarding ions may etch the newly formed Ti–N-based compounds and expose the surface beneath, which is more prone to diffusion of nitrogen. In this area, plasma nitriding techniques, especially radio frequency nitriding, give very good results in terms of surface hardness and the depth of increased hardness region. The authors emphasize the positive influence of the radio frequency electric field on the increased diffusion of nitrogen in titanium matrix [12,27].

### 3.3. Coefficient of Friction and Wear Rate

**Figure 5:**
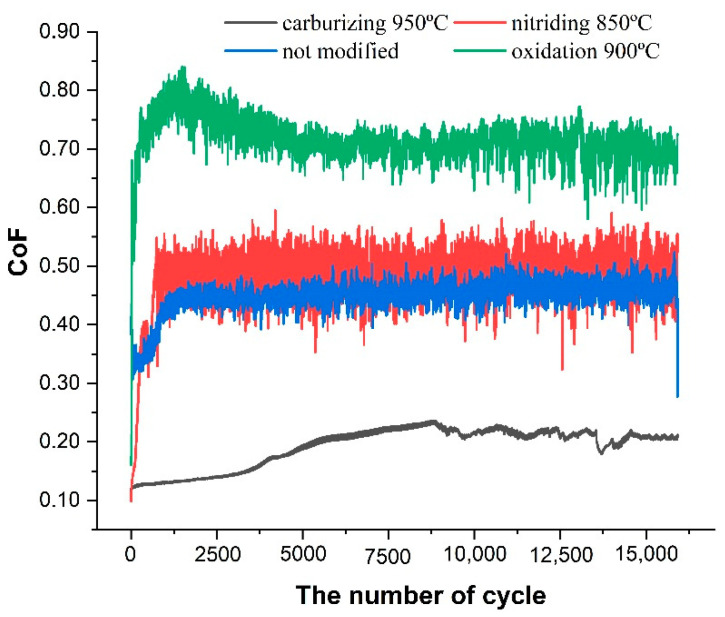
Coefficient of friction of the Ti-6Al-4V titanium alloy before and after thermo-chemical treatment.

The registered trends of CoF (Figure 5) both for modified and unmodified Ti-6Al-4V samples sliding against a ZrO_2_ ceramic counterbody show that the lowest values of coefficient of friction were obtained for carburized substrates. In this case, the coefficient of friction slightly increases within the first 50% of the test, and then it oscillates around a constant value of 0.2. This result confirms the positive effect of carburizing on lowering and increasing the stability of the coefficient of friction of titanium alloys [19]. Other surface modifications resulted in an increase in CoF as compared to the unmodified sample for which the value of CoF was about 0.47. For the nitrided sample, the resulting coefficient of friction oscillates around 0.5. A similar result was reported in [30,31]. Moreover, the authors of ref. [32] attributed this fact to the increased brittleness of the nitride layer and formation of hard abrasive debris. In the case of the oxidized Ti-6Al-4V, CoF reaches the value of 0.7. Among all the applied thermochemical treatment methods, the most visible is the increase in CoF of oxidized substrates. This phenomenon is probably related to a high chemical affinity of the substrate and the counterbody. When combined with ZrO_2_ ceramics, it possibly produces temporary adhesive TiO_x_–ZrO_2_ interactions which may be the reason of the increased value of CoF. Similar observations were reported in [33] for a ceramic-based SiO_x_–DLC layer cooperating with a ZrO_2_ counterbody and in [34] for an Al_2_O_3_ counterbody This, in turn, may indicate that for most friction pairs that include ionically bonded materials, this phenomenon is possible.

**Figure 6:**
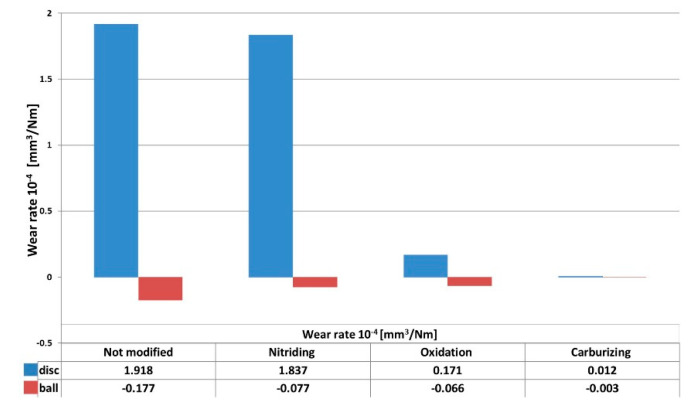
Wear rates of Ti6Al4V and ZrO_2_ counterbody: not modified, after nitriding, after oxidation, after carburizing.

After the tribological tests, the substrates and counterbodies were analyzed in terms of the quantitative and qualitative determination of the wear. As shown in Figure 6, the best wear resistance characterized the samples treated by carburizing and oxidation processes. The registered values of wear rate were 0.12 × 10^−5^ and 1.17 × 10^−5^ mm^3^/Nm, respectively. The obtained results coincide with the literature data describing these treatments often with use of different modification techniques [35,36,37].

It is worth noting that the oxidized sample showed a significant reduction in wear, despite the highest value of the friction coefficient. In the case of this sample, similarly to the unmodified alloy, the adhesive character of wear dominates, which is the reason for the high value of the coefficient of friction. On the other hand, the oxidation process leads to the greatest increase in the hardness of the substrate, which results in significantly reduced wear.

**Figure 7:**
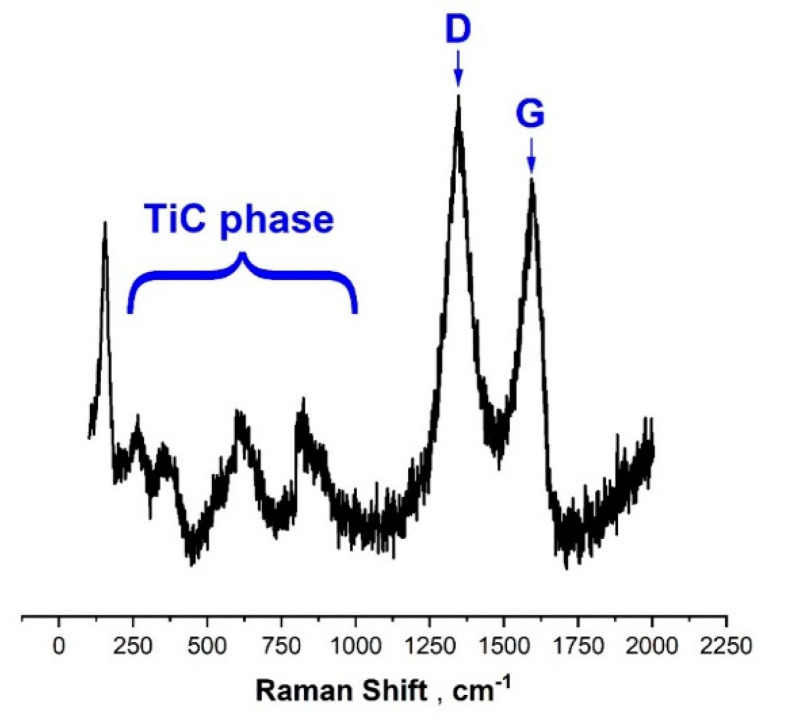
Raman spectra on the surface of Ti-6Al-4V simple after carburizing.

Comparing the wear rates of ZrO_2_ counterbodies, it can be concluded that for both nitriding and oxidation processes, the wear rate of the counterbody is similar and stays in the middle between not modified and carburized Ti-6Al-4V alloy. Nevertheless, its value is more than two times lower as compared to the test with the bare Ti-6Al-4V substrate. The lowest values of wear rate of the counterbody were registered for the carburized substrates. This phenomenon is closely related to the formation of carbon deposit on the surface of samples, which acts as a solid lubricant. This hypothesis was confirmed by the results of Raman spectroscopy. In Figure 7 is presented a typical Raman spectrum of carburized Ti-6Al-4V sample. The spectra show clear bands around 1350 and 1550 cm^−1^, with a rather high I_D_/I_G_ intensity index characteristic for amorphous carbon coatings with a relatively high concentration of sp^2^ hybridized bonds [38,39]. Moreover, within the wavenumber range between 200 and 800 cm^−1^, additional bands attributed to hypo-stoichiometric or defective TiC phases may be distinguished [40,41].

**Figure 8:**
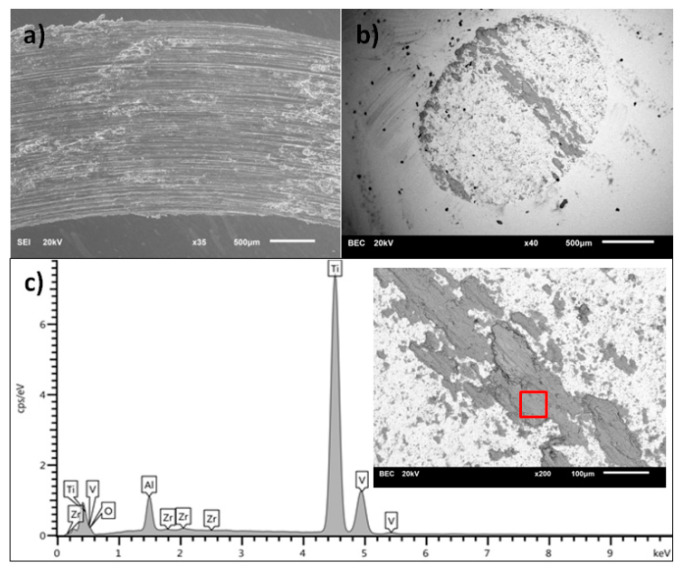
(**a**) Wear track on the surface of Ti-6Al-4V sample, (**b**) wear scar on the ZrO_2_ ball surface, (**c**) the EDS analysis of the surface of ZrO_2_ counterbody (inset with square shows the place of analysis).

**Figure 9:**
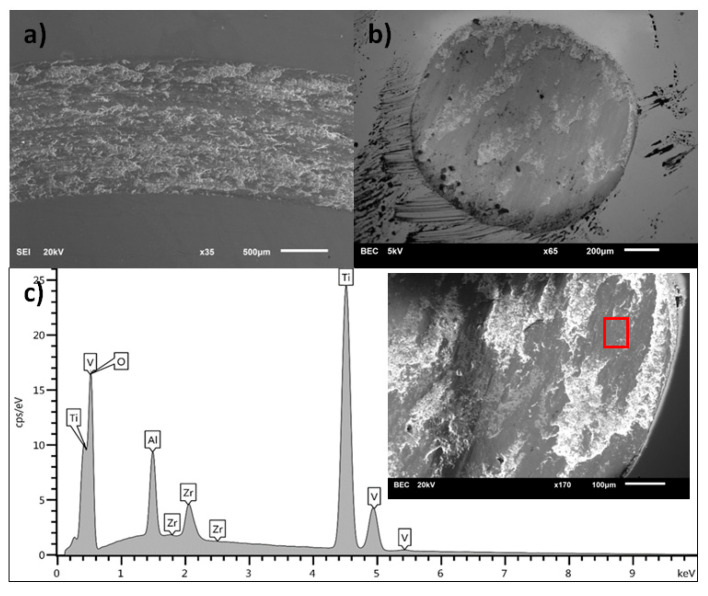
(**a**) Wear track on the surface of oxidized Ti-6Al-4V sample, (**b**) wear scar on the ZrO_2_ ball surface, (**c**) the EDS analysis of the surface of ZrO_2_ counterbody (inset with square shows the place of analysis).

**Figure 10:**
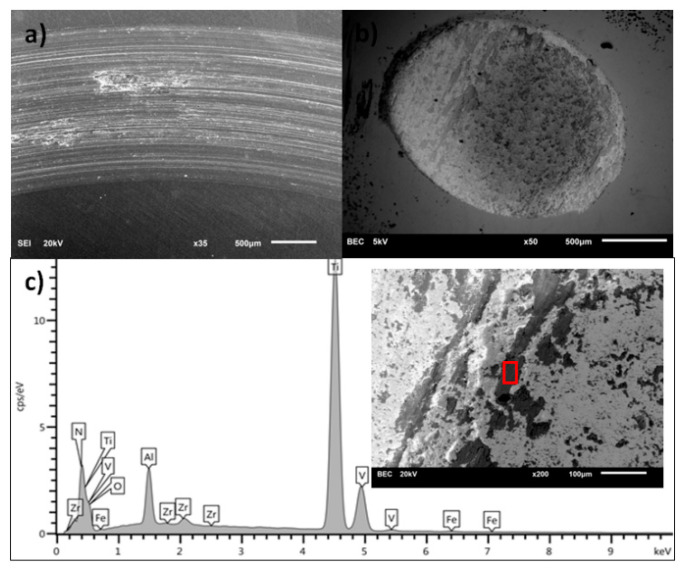
(**a**) Wear track on the surface of nitrided Ti-6Al-4V sample, (**b**) wear scar on the ZrO_2_ ball surface, (**c**) the EDS analysis of the surface of ZrO_2_ counterbody (inset with square shows the place of analysis).

**Figure 11:**
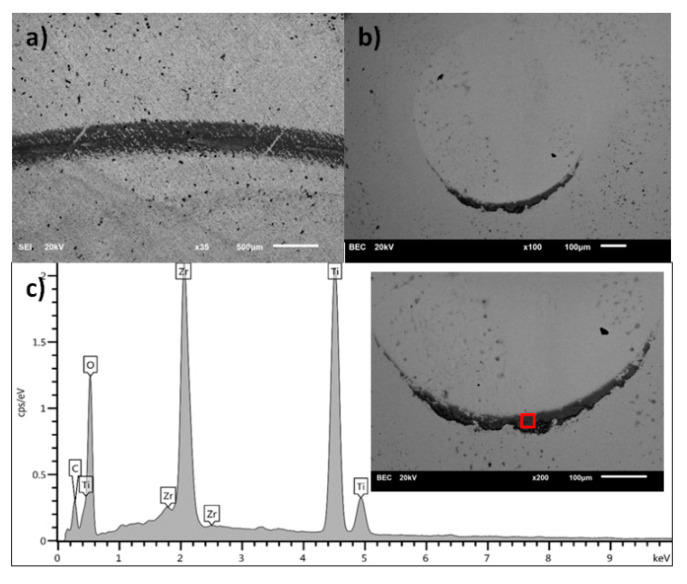
(**a**) Wear track on the surface of carburized Ti-6Al-4V sample, (**b**) wear scar on the ZrO_2_ ball surface, (**c**) the EDS analysis of the surface of ZrO_2_ counterbody (inset with square shows the place of analysis).

Figure 8, Figure 9, Figure 10 and Figure 11 present the surface morphology of the wear tracks of samples after the tests. Additionally, the images of wear scars of the counterbodies and corresponding results of EDS analysis are given. Figure 8 shows the wear track of Ti-6Al-4V alloy in combination with a ZrO_2_ ball. Clear evidence of the adhesive wear is visible in the form of noticeable amounts of the substrate materials in the wear scar on the counterbody, also confirmed by EDS. This effect was also discussed by other authors [34,37]. Interestingly, the described wear character is characteristic only for the combination of Ti-6Al-4V/ZrO_2_ and is not applicable in the case of other ceramic counterparts [34].

The nature of the wear of titanium alloy substrates after the nitriding and oxidation process is abrasive-adhesive. The substrate material is transferred to the counterbody. The exception is the carburizing process. After this modification, the nature of the wear is typically abrasive, with clear wear scars defining the direction of motion. In this case, the EDS analysis clearly shows the traces of carbon on the surface of the counterbody. This, in turn, explains the trend of friction coefficient for carburized samples, where the most uniform and stable values of CoF were registered. It is commonly known that diamond-like carbon (DLC) layers, due to the formation of a transfer layer which acts as a solid lubricant, noticeably decrease the wear of the counterbody [33].

To sum up, the tribological tests revealed that the lowest values of the coefficient of friction were registered for samples modified by carburizing. The same samples presented a noticeably lower value of the wear rate, which was two orders of magnitude lower compared to the unmodified substrate. Samples treated by plasma oxidation presented one order of magnitude higher values of wear rate as compared to the carburized ones and the highest value of the coefficient of friction. In the case of nitrided samples, no noticeable changes in the tribological parameters were found in comparison to the bare substrate. Wear track analysis of carburized surfaces revealed pure abrasive wear character, whereas for the other surfaces, the wear mechanism had an abrasive and adhesive nature.

### 3.4. Corrosion Tests

Table 1 presents the mean values of corrosion potential E_cor_, polarization resistance R_p_, corrosion current i_cor_, and corrosion rate CR, determined on the basis of the electrochemical studies.

Based on presented results, it can be seen that each of the applied surface treatment procedures resulted in significant increase in the corrosion potential. This effect is noticeable especially for oxidized and carburized samples, whose E_cor_ values are approx. 0.36 V higher when compared to the unmodified Ti-6Al-4V samples. Corrosion potential of the nitrided samples is also higher, but its high standard deviation value makes it difficult to compare with other samples. Nevertheless, the increase in E_cor_ values is attributed to the change in the surface composition of the samples, and it confirms the presence of new phases on the substrate/solution boundary for every type of treatment. As demonstrated above, the surface of the oxidized sample is coated by a thick and dense TiO_x_ passive layer, nitrided samples are coated by a very thin TiN layer, while in the case of the carburized sample, the coating consists mainly of a TiC layer, which is thick and porous. These new phases determine the E_cor_ values for modified Ti-6Al-4V samples.

More detailed information about corrosion resistance of the prepared samples is provided by the analysis of R_p_, i_cor_ and CR values presented in Table 1. Since higher polarization resistance and lower corrosion rate provide a better corrosion resistance of the material, the most corrosion-resistant surface was obtained by means of the plasma oxidation procedure. In this case, the R_p_ value is approx. three times higher, and the corresponding CR value is approx. three times lower as compared to the unmodified titanium alloy. Conversely, the opposite effect was observed in the case of carburized samples—in this case, the R_p_ value is the lowest one and the CR value is the highest one. Therefore, it can be concluded that the plasma oxidation procedure increased the corrosion resistance of studied Ti-6Al-4V alloy, whereas carburizing procedure decreased its corrosion resistance. However, taking into account that in the case of carburized samples, apart from the TiC layer on the surface, the presence of carbon deposit was also found (see Figure 7), it should be assumed that the increased values of the corrosion current and corrosion rate result not from the poor protective properties of the TiC layer, but may be related either to its high porosity or the presence of a carbon deposit. No significant changes in corrosion resistance were observed after the nitriding procedure.

**Figure 12:**
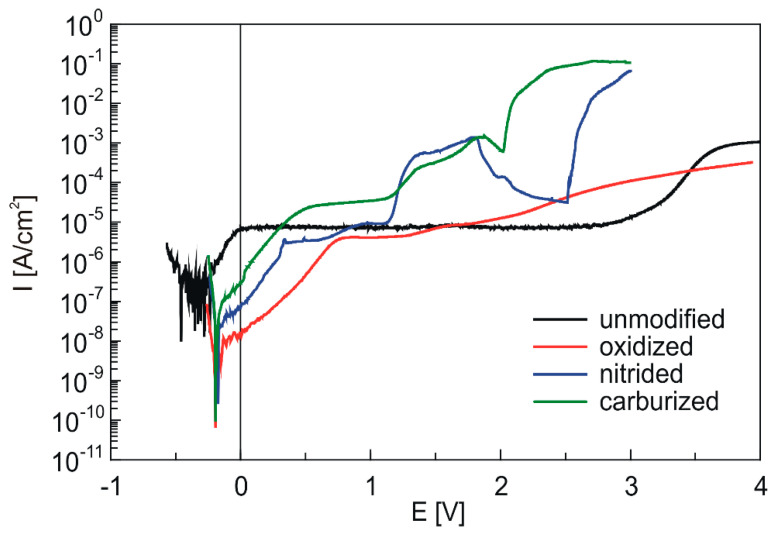
Anodic polarization curves in PBS for the titanium alloy Ti-6Al-4V after different thermo-chemical treatments (scan rate 1 mV/s).

Figure 12 shows the potentiodynamic polarization curves gathered in PBS solution for the unmodified, oxidized, nitrided, and carburized Ti-6Al-4V samples. These potentiodynamic curves, registered in wide anodic potential range, are very helpful to evaluate the resistance of tested materials to pitting corrosion. The characteristics obtained for untreated Ti-6Al-4V alloy with wide passive low-current range (passive current density of 7–8 μA/cm^2^) is typical of passive material with protective oxide layer [42].

The characteristics registered for samples after surface modification differ in the course depending on the modification procedure. For samples after oxidation, the active range of characteristics in which the Tafel relationship is satisfied reaches higher potential values and lower current values, whereas the further course of the characteristic is also typical for samples in the passive state due to the presence of a TiO_x_ layer. No significant oxidation or rapid increase in current associated with pitting corrosion is observed when the potential is swept to as high as 4 V. In addition, the analysis of the surface of the samples after polarization did not reveal any corrosion damage. In contrast, the course of potentiodynamic curves registered for Ti-6Al-4V samples modified by nitriding and carburizing procedures is different. For these modifications, the Tafel region appears at higher currents, revealing the higher electroactivity of these samples when compared to samples after oxidation. Moreover, for both nitrided and carburized samples, the anodic peaks are observed at a potential range of 1.1–2.0 V. These anodic peaks are related to electrooxidation of TiN and TiC layer formed as a result of nitriding and carburizing processes, respectively (see Section 3.1). Based on potentiodynamic curves, it can also be stated that thermo-chemical treatments such as nitriding and carburizing enhance Ti-6Al-4V alloy’s tendency towards pitting corrosion in PBS. Since this tendency can be estimated on the basis of pitting potential E_pit_ values, this parameter was determined for each sample from its potentiodynamic curve, as a potential value at which anodic current density intensively increased. It was estimated that pitting corrosion started at ca. 2.0 V in the case of carburized samples and at 2.5 V for nitrided samples. Taking into account the E_pit_ values, it can be stated that nitrided samples are relatively more pitting corrosion-resistant than carburized ones. However, it should be emphasized that despite the deteriorated pitting resistance of Ti-6Al-4V alloy after the carburizing and nitriding processes, it is still more pitting-resistant than AISI 316 stainless steel; according to the literature [43,44,45], AISI 316 in chloride-containing solutions undergoes pitting at potentials of 0.4–0.6 V, which are much lower than the pitting potentials determined for carburized and nitrided Ti-6Al-4V samples.

**Figure 13:**
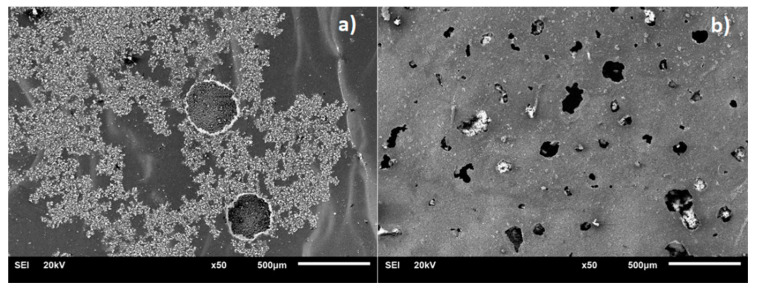
SEM images of corrosion damage formed on (**a**) nitrided and (**b**) carburized Ti6Al4V alloy samples as a result of potentiodynamic polarization.

Post-polarization microscopic observations of the surfaces revealed the presence of corrosion damage for both nitrided and carburized samples (Figure 13). However, the pits observed on both types of samples surfaces were different in shape. In the case of carburized samples, pits were of irregular shape, and were relatively deeper than in the case of nitrided ones.

**Figure 14:**
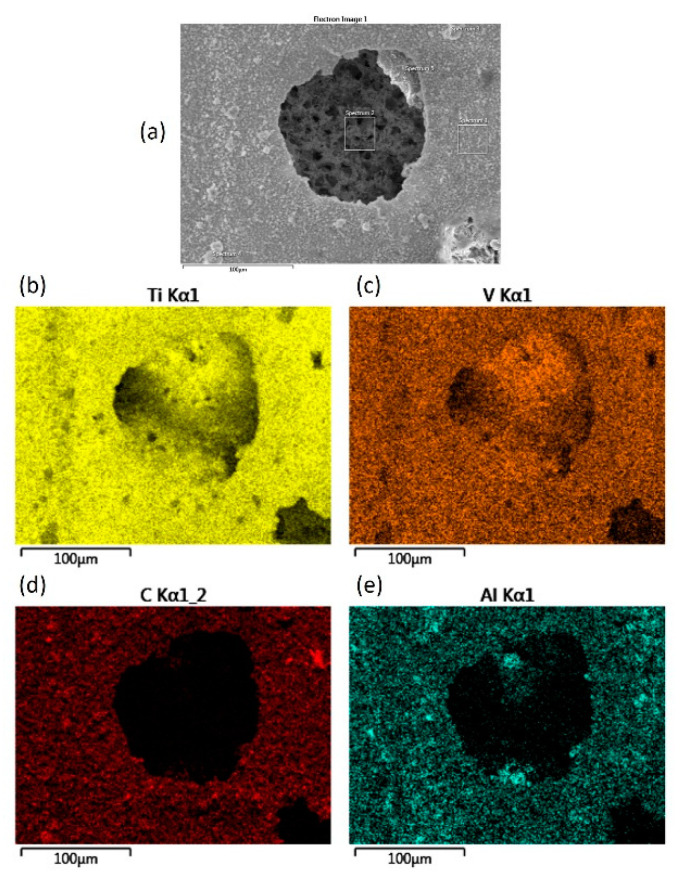
EDS element distribution map of corrosion pit formed on the surface of carburized sample: (**a**) secondary electron image of studied area, (**b**) titanium distribution map in tested area (**c**) vanadium distribution map in tested area, (**d**) carbon distribution map in tested area (**e**) aluminum distribution map in tested area.

The elemental mapping performed with the use of EDS (results presented in Figure 14) clearly shows the distribution of the main alloy elements inside and outside the pit, formed on the carburized sample. These elemental maps indicate that there is no aluminum and carbon inside the pit, therefore it can be stated that in this case, the pitting process occurs with dissolution of aluminum.

To sum up, the applied thermo-chemical treatments influenced the corrosion properties of Ti-6Al-4V alloy. The best corrosion properties were shown in samples after the oxidizing procedure, while the worst were carburized samples. Observed differences in corrosion resistance of tested samples are closely related to their different final microstructure, as well as the thickness, structure and composition of the compound layer on the top. The plasma oxidation process resulted in the formation of a Ti-O based passive layer, which is corrosion-resistant. On the other hand, a thick and very porous compound layer on the top of carburized Ti-6Al-4V alloy, mainly composed of TiC, is responsible for its weak corrosion resistance in PBS. Nevertheless, when compared to stainless steel, it is still more pitting corrosion-resistant and possesses increased hardness and wear resistance.

The research included in this work shows that the oxidation and carburization treatments significantly changed the tribological properties of the titanium alloys, each of them in a different way. Glow discharge oxidation allowed for the highest material hardening and improved corrosion resistance as compared to the unmodified alloy. However, it also caused a significant increase in coefficient of friction, with a relatively low wear rate. However, high frictional resistance resulting from the adhesive character of wear seems to be a clear disadvantage of this treatment. On the other hand, it can be an excellent pre-treatment step for titanium alloys, subsequently modified by low-friction coatings, such as DLC. In turn, carburizing gave a clear improvement in tribological properties, both the coefficient of friction and the wear rate. This may indicate that this treatment can give good results without the need for coatings. Nevertheless, at the same time, it should be noted that carburizing leads to a decrease in the corrosion resistance of the titanium alloy. In this case, further investigation whether the corrosion resistance of the carburized alloy is within the limits acceptable for medical applications is necessary. The weakest effects were achieved by the gas nitriding. No significant benefits were noticed in his case. In the light of the literature review, it seems that better results could be obtained with use of the glow discharge nitriding technique, which was described in an earlier part of the work.

## 4. Conclusions

The Ti-6Al-4V titanium alloy was thermo-chemically treated by means of oxidation, carburizing and nitriding techniques. The goal of the research was to improve the tribological properties of titanium alloy while keeping its good corrosion resistance. The process parameters of particular modifications were matched, based on the results of tribological and corrosion features, so as to obtain their optimal combination. The obtained samples were analyzed in terms of mechanical, tribological and corrosion properties. In the case of all modifications, the appearance of a surface layer with modified thickness and properties was observed. The greatest hardness (above 12 GPa) and depth of the increased hardness region (above 60 micrometers) were characteristic for plasma oxidized Ti-6Al-4V sample. For carburizing, these values were 8 GPa and 20 µm, respectively. The lowest values of hardness and depth of modification were definitely obtained for the nitrided sample and were equal to 6 GPa and less than 20 µm, respectively.

In conclusion, the weakest results in all types of tests were obtained for nitrided samples. Besides the lack of improvement in tribological properties (CoF = 0.5, wear rate: 1.837·10^−4^ mm^3^/Nm), a deterioration in the corrosion resistance was observed. The best results in terms of tribological properties were obtained for carburized surfaces (CoF = 0.2, wear rate: 0.012·10^−4^ mm^3^/Nm); however, this was in favor of a significant decrease in the resistance against corrosion. This decrease, however, did not reach critical values, and taking into account the very good results of tribological studies, this type of modification seems to be highly interesting in frictional applications. However, further research is necessary to confirm this thesis. Good results have also been obtained for the oxidized surfaces that have improved in both corrosion and wear. However, the disadvantage in this case in the high coefficient of friction (CoF = 0.7, wear rate: 0.171·10^−4^ mm^3^/Nm).

Based on the presented results, it should be stated that satisfactory results were obtained for carburizing and oxidation treatments. However, in the case of oxidation, due to the high coefficient of friction, this treatment should not be the end result of surface modification intended for tribological applications. This procedure can be perfect as a pre-treatment aimed at strengthening the substrate surface before the synthesis of low-friction coatings. On the other hand, an unacceptable result was obtained when gas nitriding was used.

## Figures and Tables

**Table 1:** Corrosion parameters of Ti-6Al-4V alloy after different thermo-chemical treatments (given as mean values ± standard deviation, n = 3).

Sample	E_cor_ [V]	R_p_ [MΩ·cm^2^]	i_cor_ [A/cm^2^]	CR [µm/year]
unmodified	−0.410 ± 0.039	0.46 ± 0.21	(6.69 ± 3.22) × 10^−8^	0.59 ± 0.28
oxidized	−0.049 ± 0.021	1.25 ± 0.20	(2.11 ± 0.31) × 10^−8^	0.19 ± 0.03
nitrided	−0.075 ± 0.063	0.41 ± 0.05	(6.49 ± 0.87) × 10^−8^	0.57 ± 0.08
carburized	−0.045 ± 0.006	0.14 ± 0.01	(1.85 ± 0.03) × 10^−7^	1.62 ± 0.03
